# Phenotypic and transcriptional response to selection for alcohol sensitivity in *Drosophila melanogaster*

**DOI:** 10.1186/gb-2007-8-10-r231

**Published:** 2007-10-31

**Authors:** Tatiana V Morozova, Robert RH Anholt, Trudy FC Mackay

**Affiliations:** 1 Department of Zoology, North Carolina State University, Raleigh, NC 27695, USA; 2 WM Keck Center for Behavioral Biology, North Carolina State University, Raleigh, NC 27695, USA; 3 Institute of Molecular Genetics RAS, Kurchatov Square, Moscow 123182, Russia; 4 Department of Genetics, North Carolina State University, Raleigh, NC 27695, USA

## Abstract

**Background:**

Alcoholism is a complex disorder determined by interactions between genetic and environmental risk factors. *Drosophila *represents a powerful model system to dissect the genetic architecture of alcohol sensitivity, as large numbers of flies can readily be reared in defined genetic backgrounds and under controlled environmental conditions. Furthermore, flies exposed to ethanol undergo physiological and behavioral changes that resemble human alcohol intoxication, including loss of postural control, sedation, and development of tolerance.

**Results:**

We performed artificial selection for alcohol sensitivity for 35 generations and created duplicate selection lines that are either highly sensitive or resistant to ethanol exposure along with unselected control lines. We used whole genome expression analysis to identify 1,678 probe sets with different expression levels between the divergent lines, pooled across replicates, at a false discovery rate of *q *< 0.001. We assessed to what extent genes with altered transcriptional regulation might be causally associated with ethanol sensitivity by measuring alcohol sensitivity of 37 co-isogenic *P*-element insertional mutations in 35 candidate genes, and found that 32 of these mutants differed in sensitivity to ethanol exposure from their co-isogenic controls. Furthermore, 23 of these novel genes have human orthologues.

**Conclusion:**

Combining whole genome expression profiling with selection for genetically divergent lines is an effective approach for identifying candidate genes that affect complex traits, such as alcohol sensitivity. Because of evolutionary conservation of function, it is likely that human orthologues of genes affecting alcohol sensitivity in *Drosophila *may contribute to alcohol-associated phenotypes in humans.

## Abstract 2

Gene-expression profiling combined with selection for genetically divergent *Drosophila *lines either highly sensitive or resistant to ethanol exposure has been used to identify candidate genes that affect alcohol sensitivity, including 23 novel genes that have human orthologs.

## Background

Alcohol abuse and alcoholism are significant public health problems throughout the world. In the United States alone, they affect approximately 14 million people at a health care cost of $184 billion per year [1].

Identifying genes that predispose to alcoholism in human populations has been hampered by genetic heterogeneity and the inability to control environmental factors, and the reliance on complex psychiatric assessments and questionnaires to quantify alcohol-related phenotypes. Despite these disadvantages, studies in ethnically defined populations have implicated alleles of alcohol dehydrogenase, aldehyde dehydrogenase, the GABA_A _receptor complex, and the serotonin 1B receptor as contributing to variation in alcohol sensitivity (reviewed in [2-5]). Recently, large scale gene expression profiling identified candidate alcohol responsive genes in human brains [6-10], including genes that encode proteins involved in myelination, neurodegeneration, protein trafficking as well as calcium, cAMP, and thyroid signaling pathways. It is, however, difficult to design large scale experiments in humans to verify causal roles for these candidate genes.

Studies in mice have provided further support for important roles of serotonin, GABA_A _and dopamine receptors as well as opioid peptides (reviewed in [11,12]) in modulating the effects of alcohol. In addition, four classes of protein kinases, PKA, PKC, PKG and Fyn kinase, have been identified as critical mediators of the effects of alcohol [13-16]. Changes in brain gene expression following exposure to alcohol have also been observed in inbred mouse strains for multiple genes associated with the Janus kinase/signal transducers and activators of transcription, the mitogen activated protein kinase pathways, and retinoic acid mediated signaling [17].

With its well annotated genome and amenability to powerful genetic manipulations, *Drosophila *presents an attractive model organism for studies on the genetic architecture of alcohol sensitivity [18,19]. Although flies do not exhibit addictive behavior according to the formal criteria for diagnosing substance abuse disorders in humans [5], alcohol sensitivity and the development of alcohol tolerance in flies show remarkable similarities to alcohol intoxication in vertebrates, suggesting that at least some aspects of the response to alcohol may be conserved across species [20]. Moreover, two-thirds of human disease genes have orthologues in *Drosophila *[21]. Exposing flies to low concentrations of ethanol stimulates locomotor activity, whereas high concentrations of ethanol induce an intoxicated phenotype, characterized by locomotor impairments, loss of postural control, sedation and immobility [22,23].

Studies to date have used mutant screens and expression profiling of flies after exposure to alcohol and after development of tolerance to identify genes associated with ethanol sensitivity in *Drosophila *[19,24-29]. An alternative strategy to discover genes affecting complex behaviors is to combine artificial selection for divergent phenotypes with whole genome expression profiling [3,30-33]. The rationale of this approach is that genes exhibiting consistent changes in expression as a correlated response to selection are candidate genes affecting the selected trait [33].

Here, we performed 35 generations of artificial selection from a genetically heterogeneous base population to derive replicate lines that are sensitive or resistant to ethanol exposure, as well as unselected control lines. We used whole genome transcriptional profiling to identify genes that are differentially expressed between the selection lines. Functional tests of mutations in 35 of the differentially expressed genes confirmed 32 novel candidate genes affecting alcohol sensitivity, including three (*Malic enzyme*, *nuclear fallout and longitudinals lacking*) that have been previously associated with alcohol sensitivity and/or tolerance in *Drosophila *[19]. A high proportion of this subset of candidate genes (72%) has human orthologues and their human counterparts are, therefore, relevant candidate genes that may predispose to alcohol sensitivity and alcohol abuse in human populations.

## Results

### Phenotypic response to artificial selection for alcohol sensitivity

**Figure 1:**
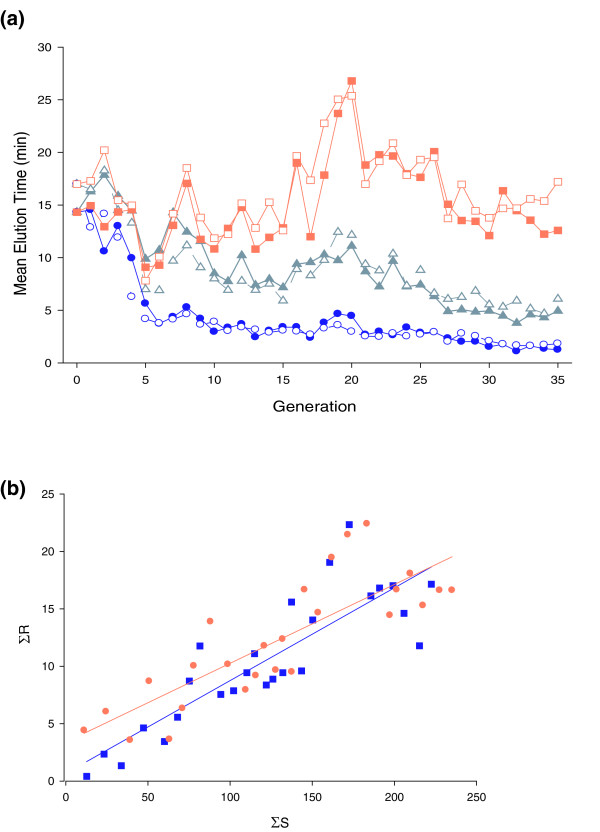
Phenotypic response to selection for alcohol sensitivity. **(a) **MET for selection lines. Resistant lines are shown as orange squares, control lines as grey triangles, and sensitive lines as blue circles. Solid lines and shapes represent replicate 1; dashed lines and open shapes denote replicate 2. **(b) **Regressions of cumulative response on cumulative selection differential for divergence between resistant and sensitive selection lines. The blue line and squares represent replicate 1; the orange line and circles denote replicate 2.

We constructed a heterogeneous base population from isofemale lines sampled from a Raleigh natural population and used artificial selection to create replicate genetically divergent lines with increased resistance (R) or sensitivity (S) to ethanol exposure. We also generated replicate unselected control (C) lines to enable us to monitor the symmetry of the response and genetic drift. Lines had established maximum divergence after 25 generations of selection. At generation 25, the mean elution time (MET) for the replicate control lines (C1 and C2) was MET = 7.4 minutes and MET = 8.8 minutes, respectively; for the replicate sensitive lines (S1 and S2), MET = 2.9 minutes and MET = 2.7 minutes, respectively; and for the replicate resistant lines (R1 and R2), MET = 17.6 minutes and MET = 19.3 minutes, respectively (Figure 1a). Thus, the R and S replicate lines diverged from each other by an average of 15.65 minutes at generation 25. The response to selection was symmetrical. Realized heritability estimates from the divergence between R and S lines over 25 generations were *h*^2 ^= 0.081 ± 0.0097 (*P *< 0.0001) and *h*^2 ^= 0.069 ± 0.0096 (*P *< 0.0001) for the respective replicates (Figure 1b). After generation 25 there was almost no response to selection. Realized heritability estimates from the divergence between R and S lines from generation 25 to 35 were *h*^2 ^= -0.056 ± 0.036 (*P *= 0.1567) and *h*^2 ^= 0.0031 ± 0.027 (*P *= 0.91) for the respective replicates.

### Correlated phenotypic responses to selection for alcohol sensitivity

Exposure to alcohol affects locomotion [22,23]. Furthermore, in human populations excessive alcohol consumption can give rise to aggressive and violent behaviors [34-36]. Alcohol sensitivity also depends on metabolic and physiological state [37-41]. In addition, exposure to alcohol results in an acute down-regulation of the expression of a group of odorant receptors and odorant binding proteins [19], which raises the question whether artificial selection for alcohol sensitivity would be associated with a reduction in olfactory ability. To assess whether the response to selection was specific for alcohol sensitivity or whether other phenotypes underwent correlated selection, we tested the selection lines for locomotion, aggression, starvation resistance, and olfactory behavior.

**Figure 2:**
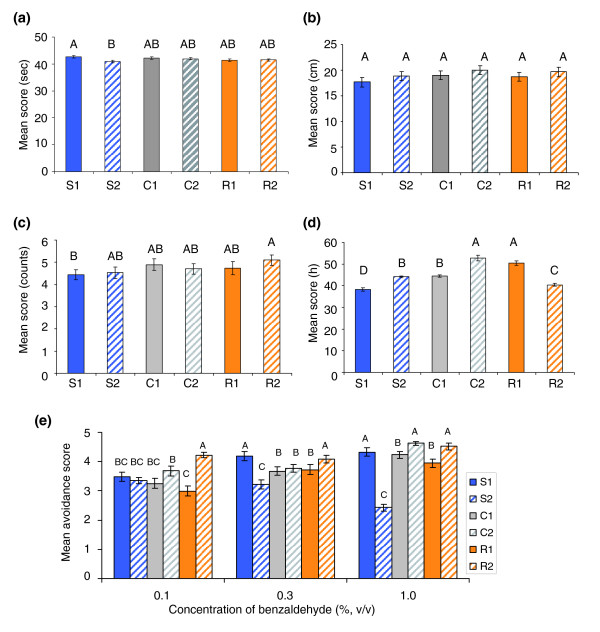
Correlated phenotypic responses to selection. Lines with the same letter are not significantly different from one another at *p *< 0.05. Resistant lines are colored orange, control lines grey, and sensitive lines blue. Solid lines and bars represent replicate 1; dashed bars and lines denote replicate 2. **(a) **Locomotor reactivity; **(b) **climbing behavior; **(c) **aggression behavior; **(d) **starvation resistance; **(e) **olfactory avoidance behavior. Error bars indicate standard errors.

We found no differences in locomotor behavior among the selection lines using either an assay for locomotor reactivity (F_2,3 _= 3.14, *p *= 0.18; Figure 2a) or a climbing assay (F_2,3 _= 1.48, *p *= 0.36; Figure 2b). The selection lines also did not differ in the number of aggressive encounters under conditions of competition for limited food (F_2,3 _= 3.10, *p *= 0.19; Figure 2c). Selection lines also did not differ in starvation resistance (F_2,3 _= 0.56, *p *= 0.64; Figure 2d). Finally, there was no correlation between alcohol sensitivity and olfactory avoidance behavior over a range of concentrations of the repellent odorant benzaldehyde (F_2,3 _= 0.40, *p *= 0.70; Figure 2e) (although there were significant differences between replicates of selection lines in avoidance response (F_3,3 _= 455.36, *p *= 0.0002), with line S2 showing reduced olfactory responsiveness). Our results, therefore, indicate that the response to selection was specific for alcohol sensitivity.

### *Alcohol dehydrogenase* gene frequencies

*Drosophila *encounters ethanol in its natural habitat, as flies feed on fermented food sources. Natural selection, at least under some environmental conditions, affects allele frequencies of the *Alcohol dehydrogenase *(*Adh*) locus, which is polymorphic for two allozymes, which differ by a single amino acid (T192K), designated Slow and Fast, based on their gel migration profile [42,43]. Fast homozygotes have a higher level of enzymatic activity than Slow homozygotes and a higher tolerance to alcohol in laboratory toxicity tests [44-46].

To assess whether differences in alcohol sensitivity in our selection lines could be attributed in part to the Slow and Fast electrophoretic alleles of *Adh *[45,47], we developed a single nucleotide polymorphism marker for this polymorphism and measured allele frequencies in our selection lines. Frequencies of the Fast allele in the replicate control lines were 0.79 and 0.24. The R1 and R2 replicate lines had Fast allele frequencies of 0.42 and 0.58, respectively. However, in both the sensitive selection lines the Slow allele was fixed. Previous studies have shown that flies homozygous for the Slow *Adh *allele are more sensitive to alcohol [46].

### Transcriptional response to selection for alcohol sensitivity

We used Affymetrix high density oligonucleotide microarrays to assess whole genome transcript abundance in three- to five-day-old flies of the selection lines at generation 25. Raw expression data have been deposited in NCBIs Gene Expression Omnibus [48] and are accessible through GEO series number (GSE 7614).

We used a stepwise procedure to analyze the data. First, we used factorial ANOVA to quantify statistically significant differences in transcript levels for each probe set on the array. Using a stringent false discovery rate [49] of *q *< 0.001, we found that 9,931 probe sets were significant for the main effect of sex, 2,612 were significant for the main effect of line, and 184 were significant for the line × sex interaction term (Additional data file 1). Only two genes that were significant for the interaction term were not significant for the main effect of line: *CG1751*, which is involved in proteolysis, and *CG12128*, which encodes a transcript of unknown function.

Next, we used ANOVA contrast statements on the 2,612 probe sets with differences in transcript abundance between selection lines to detect probe sets that were consistently up- or down-regulated in replicate lines [31]. We identified 2,458 probe sets (13% of the total probe sets on the microarray) that differed between the selection lines when pooled across replicates (Additional data file 2).

**Figure 3:**
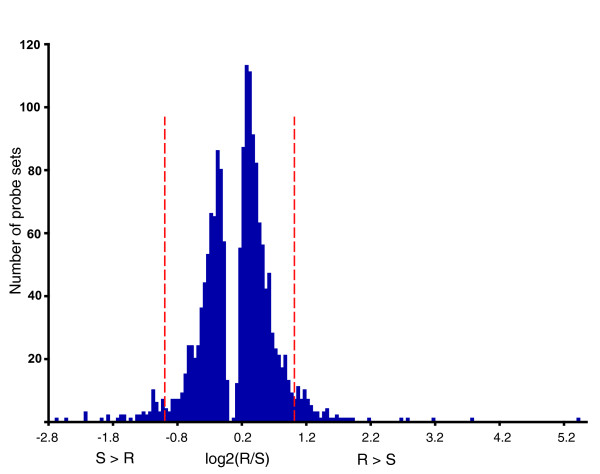
Histogram showing the frequency of relative fold-change in probe sets with significant differences in transcript abundance between resistant (R) and sensitive (S) selection lines, pooled over sexes. The vertical dashed red lines demarcate two-fold changes in transcript abundance.

Among these 2,458 probe sets, 1,572 were divergent between resistant and control lines, 1,617 between sensitive and control lines, and 1,678 between resistant and sensitive selection lines. Although the transcriptional response to selection for alcohol sensitivity was widespread, the magnitudes of the changes in transcript abundance were relatively small, with the vast majority of probe sets showing less than two-fold changes in abundance (Figure 3). In fact, only 121 probe sets showed larger than two-fold differences in transcript abundance. Among these probe sets 37 have not been annotated; 14 encode genes involved in defense response and response to stress, including *Defensin*, *Attacin-A*, *Lysozyme P*, *Immune induced molecules 1*, *10*, and *23*, and *Metchnikowin*; and 12 probe sets that encode gene products involved in carbohydrate metabolism (*sugar transporter 1*, *Mitogen-activated protein kinase phosphatase 3*, *CG9463*, *CG14959 CG10725*, *CG10924*, *Lysozyme P*) (Additional data files 3 and 4).

### Categories of genes with differential transcript abundance among sensitive and resistant lines

Probe sets with altered transcript abundance between selection lines fell into all major biological process and molecular function Gene Ontology (GO) categories (Additional data files 5 and 6). We used χ^2 ^tests to determine which categories were represented more or less frequently than expected by chance, based on their representation on the microarray. One interpretation of these analyses is that over-represented GO categories contain probe sets for which transcript abundance has responded to artificial selection, whereas under-represented GO categories contain probe sets for which transcript abundance is under stabilizing natural selection [31]. Highlights of the transcriptional response to artificial selection for alcohol sensitivity for probe sets differentially expressed between resistant and sensitive selection lines are given in Table 1. For example, the resistant lines are enriched for up-regulated genes affecting responses to chemical stimulus (including response to toxin and pheromone), extracellular transport, and lipid metabolism; while the sensitive lines are enriched for up-regulated genes affecting alcohol metabolism, defense response, electron transport, catabolism, and lipid and carbohydrate metabolism. Transcripts in the 'response to toxin' GO category are over-represented in both sensitive and resistant lines, but the magnitude of over-representation is higher for resistant lines (*p *= 4.19E-7, compared to *p *= 0.029 for sensitive lines. GO categories for lipid metabolism are notably over-represented in sensitive lines (*p *= 1.29E-11, compared to *p *= 1.2E-04 for resistant lines).

**Table 1:** Differentially over-represented biological function GO categories between resistant (R) and sensitive (S) lines

R > S	S > R
Response to abiotic stimulus 1.80E-06*	**Alcohol metabolism 4.00E-03**
Response to chemical stimulus 1.26E-06	Response to toxin 2.90E-02
Response to toxin 4.19E-07	**Response to biotic stimulus 4.32E-05**
Response to pheromone 2.10E-08	Defense response 2.13E-05
Chemosensory behavior 8.00E-04	Immune response 1.00E-04
**Extracellular transport 2.07E-08**	**Electron transport 2.88E-05**
**Lipid metabolism 8.0E-05**	**Lipid metabolism 2.99E-09**
Cellular lipid metabolism 1.20E-04	Cellular lipid metabolism 1.29E-11
Phospholipid metabolism 2.30E-03	Organic acid metabolism 2.37E-07
Steroid metabolism 2.00E-05	Steroid metabolism 9.80E-4
	Fatty acid metabolism 1.56E-10
	**Catabolism 1.53E-05**
	Cellular catabolism 1.08E-06
	**Carbohydrate metabolism 5.20E-05**

*p values were calculated from χ^2 ^tests, estimating which categories were represented more frequently than expected by chance, based on their representation on the microarray.

These GO categories correlate well with GO categories that were over-represented during the acute response to a single exposure to ethanol [19], which also resulted in extensive changes in transcript abundance for chemosensory behavior, response to chemical stimulus, and response to toxin.

### Pleiotropy

Changes in expression of transcripts during artificial selection for locomotor reactivity, aggression, and alcohol sensitivity [32,33] each encompass a significant percentage of the genome, implying extensive pleiotropy. We found that the transcriptional response to selection for alcohol sensitivity results in changes in expression of over 2,600 probe sets (approximately 14% of the genome) between the selection lines at a stringent false discovery rate of *q *< 0.001. Similarly, transcript abundance of over 1,800 probe sets evolved as a correlated response to selection for increased and decreased levels of locomotor reactivity [33] and expression of over 1,500 probe sets changed during selection for high and low levels of aggressive behavior [32]. Since these studies used the same initial base population, we could assess overlap in transcripts with altered expression between our selection lines and data from previous studies with lines selected for locomotor reactivity and aggression.

We used χ^2 ^tests to assess whether we observed more common differentially regulated probe sets than expected by chance. We found 727 probe sets in common between lines selected for alcohol sensitivity and locomotor reactivity, (χ_1_^2 ^= 883, *p *<< 0.0001); 474 probe sets in common between lines selected for aggressive behavior and locomotor reactivity (χ_1_^2 ^= 731, *p *<< 0.0001); and 674 probe sets in common between lines selected for alcohol sensitivity and aggressive behavior (χ_1_^2 ^= 986.1, *p *<< 0.0001). The transcript abundance of 307 genes was altered as a correlated response to selection for all three behaviors (χ_1_^2 ^= 3928.87, *p *<< 0.0001).

GO categories that were significantly over-represented among these 307 genes include lipid metabolism (*p *= 2.2E-16), electron transport (*p *= 1.2E-7), response to chemical stimulus (*p *= 6.1E-5), carbohydrate metabolism (*p *= 9.4E-5) and generation of precursor metabolites and energy (*p *= 8.4E-7). These genes included 17 members of the cytochrome P450 family and additional genes involved in defense response and/or response to toxin (*Glutathione S transferases D9*, *E1 *and *E5*; *Immune induced molecule 10*, *Cbl*, *UDP-glycosyltransferase 35b*, *Juvenile hormone epoxide hydrolase 1 *and *2*, *Lysozyme P *and *Peroxiredoxin 2540*; Additional data files 7 and 8). Members of this group of 307 genes appear to represent a common group of environmental response genes.

### Functional tests of candidate genes

**Figure 4:**
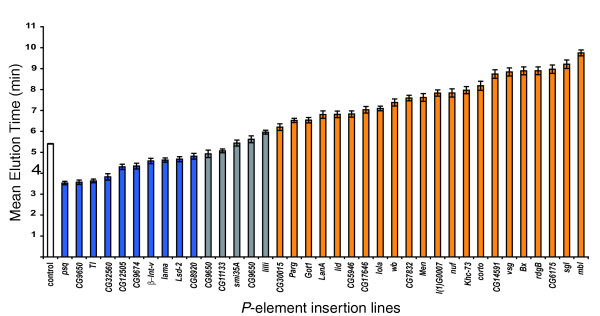
MET of lines containing *P*-element insertions in candidate genes. The white bar denotes the *Canton S *B co-isogenic control line; grey bars indicate lines with MET not significantly different from the control; blue bars indicate lines significantly sensitive to alcohol vapor to compare with the control (*p *< 0.05); and orange bars indicate lines significantly resistant than the control (*p *< 0.05). Error bars indicate standard errors.

To validate our premise that transcriptional profiling of artificial selection lines can identify candidate genes that contribute to the trait that responds to selection, we measured alcohol sensitivity of 45 independent *P[GT1]*-element insertion lines corresponding to 35 candidate genes [50,51]. These candidate genes are involved in diverse biological processes, including carbohydrate metabolism (*Malic enzyme*, *Poly(ADP-ribose)glycohydrolase*, *CG9674*), regulation of transcription (*little imaginal discs*, *pipsqueak*, *lilliputian*, *longitudinals lacking*, *CG9650*), nervous system development (*Beadex*, *Laminin A*, *longitudinals lacking*, *muscleblind*, *smell impaired 35A*), lipid metabolism (*retinal degeneration B*, *sugarless*, *CG17646*) and signal transduction (*βν integrin*, *Laminin A*, *sugarless*, *wing blister*, *CG32560*). Five of the candidate genes encode predicted transcripts of unknown function (*lamina ancestor*, *CG11133*, *CG30015*, *CG14591 *and *CG6175*). Overall, 33 (73%) of the *P[GT1]*-element insertion lines exhibited significant differences in alcohol sensitivity compared to co-isogenic *Canton S *(B) control at *p *< 0.05, and for 19 of these lines (58%) statistically significant differences from the control survived Bonferroni correction for multiple tests (Table 2, Figure 4). Remarkably, *P*-element insertions implicate 32 out of 35 genes in alcohol sensitivity. *P*-element mutants in *Beadex*, *corto*, *Glutamate oxaloacetate transaminase 1*, *Kinesin-73*, *Laminin A*, *lethal (1) G0007*, *little imaginal discs*, *longitudinals lacking*, *Poly(ADP-ribose) glycohydrolase*, *Malic enzyme*, *muscleblind*, *nuclear fallout*, *retinal degeneration B*, *sugarless*, *visgun*, *wing blister*, *CG6175*, *CG14591*, *CG7832*, *CG17646*, *CG5946 *and *CG30015 *were more resistant to ethanol exposure than the control. In contrast, mutants for *βν integrin*, *lamina ancestor*, *Lipid storage droplet-2*, *pipsqueak*, *Toll*, *CG9650*, *CG32560*, *CG12505 *and *CG9674 *were more sensitive to ethanol exposure than the control. Three of these *P*-element insertion lines with transposon insertions at *Malic enzyme*, *nuclear fallout *and *longitudinals lacking *were previously implicated in alcohol sensitivity and/or tolerance in *Drosophila *[19].

**Table 2:** Functional tests of candidate genes

Line	Gene name	MET (SE)	*p *value	Human orthologue	Biological function
*Canton S B*	Control	5.4 (0.01)	NA	NA	NA
**BG02818** BG02327	*pipsqueak (psq)*	3.5 (0.08) 5.75 (0.22)	<0.0001 0.1776	NA	Regulation of transcription, DNA-dependent
**BG02845**	*Toll (Tl)*	3.6 (0.09)	<0.0001	toll-like receptor 4*	Defense response, immune response, Toll signaling pathway
**BG02317** BG01705 BG02624	*CG9650*	3.6 (0.10) 4.9 (0.18) 5.6 (0.16)	<0.0001 0.1118 0.6381	B-cell lymphoma/leukemia 11A*	Regulation of transcription from RNA polymerase II promoter, nucleic acid binding
BG02522	*CG32560*	3.8 (0.15)	<0.0032	DAB2 interacting protein*	Ras protein signal transduction, G-protein coupled receptor, MAPKKK cascade
BG01371	*CG12505*	4.3 (0.13)	0.0012	NA	Nucleic acid binding
BG02560	*CG9674*	4.3 (0.14)	0.0042	NA	Carbohydrate metabolism, amino acid biosynthesis
BG02210 BG02523	*lamina ancestor (lama)*	4.6 (0.10) 5.7 (0.36)	0.0014 0.6441	NP_775813, novel gene	Unknown
BG01037	*β*^*ν *^*integrin (βInt-ν)*	4.6 (0.13)	0.0014	NA	Signal transduction, defense response
BG02812 BG02830	*Lipid storage droplet-2 (Lsd-2)*	4.7 (0.12) 5.52 (0.23)	0.0083 0.7909	*Adipose differentiation-related protein*	Lipid transport, sequestering of lipid
BG02518	*CG8920*	4.7 (0.11)	0.0025	*Tudor domain containing protein 7*	Nucleic acid binding
BG00987	*smell impaired 35A (smi35A)*	5.4 (0.14)	0.9859	*dual-specificity tyrosine-(Y)-phosphorylation regulated kinase 4*	Olfactory behavior, response to chemical stimulus, nervous system development
BG01008	*CG11133*	5.6 (0.09)	0.6816	NA	Unknown
BG02207 BG2034	*lilliputian (lilli)*	5.8 (0.11) 6.0 (0.28)	0.0905 0.1549	Fragile X mental retardation 2 protein*	Regulation of transcription, DNA-dependent
BG02114 BG01509	*CG30015*	6.2 (0.17) 5.6 (0.31)	0.0120 0.4924	NA	Unknown
BG02055	*little imaginal discs (lid)*	6.4 (0.11)	0.0283	*Jumonji*, *AT rich interactive domain 1A*	Regulation of transcription, DNA dependent
BG01081	*Glutamate oxaloacetate transaminase 1 (Got1)*	6.5 (0.10)	0.0048	*Glutamic-oxaloacetic transaminase 1*	Amino acid metabolism, biosynthesis
BG01013	*Poly(ADP-ribose) glycohydrolase (Parg)*	6.5 (0.28)	0.0137	Poly(ADP-ribose) glycohydrolase*	Carbohydrate metabolism, glycolysis
BG01389	*Laminin A (LanA)*	6.8 (0.15)	0.0020	*laminin*, *alpha-5*	Proteolysis, signal transduction, central nervous system development
**BG02420**	*CG5946*	6.9 (0.15)	<0.0001	Cytochrome b5 reductase 3*	Fatty acid desaturation, cholesterol metabolism
**BG01144**	*CG17646*	7.0 (0.16)	0.0002	NA	Lipid metabolism, nucleotide binding
**BG02731** BG02501	*longitudinals lacking (lola)*	7.1 (0.11) 5.8 (0.31)	<0.0001 0.5457	*Zinc finger and BTB domain containing protein 3*	Regulation of transcription from RNA polymerase II promoter, nervous system development
**BG02276**	*CG7832*	7.6 (0.13)	<0.0001	NA	Protein binding
**BG02365**	*Malic enzyme (Men)*	7.7 (0.24)	<0.0001	*Malic enzyme 1*, *NADP(+)-dependent*	Malate metabolism, carbohydrate metabolism
**BG02180**	*nuclear fallout (nuf)*	8.1 (0.18)	<0.0001	*RAB11 family interacting protein 4*	Protein binding, actin cytoskeleton reorganization
**BG01342**	*Kinesin-73 (Khc-73)*	8.1 (0.21)	<0.0001	*kinesin family member 13A*	Protein targeting, exocytosis
BG02405 BG00525	*corto*	8.2 (0.21) 5.2 (0.21)	0.0086 0.2390	NA	Cell cycle, RNA polymerase II transcription factor activity, protein binding
**BG02128**	*lethal (1) G0007 (l(1)G0007)*	8.3 (0.13)	<0.0001	*DEAH-box protein 38*	Nuclear mRNA splicing, via spliceosome; nucleic acid binding
**BG01672**	*CG14591*	8.7 (0.20)	<0.0001	*Transmembrane protein 164*	Unknown
**BG01989**	*visgun (vsg)*	8.8 (0.19)	<0.0001	Transmembrane protein 123*	Learning and/or memory, olfactory learning
**BG00291**	*retinal degeneration B (rdgB)*	8.9 (0.19)	<0.0001	*phosphatidylinositol transfer protein*, *membrane-associated 2*	Lipid metabolism, sensory perception of smell, calcium ion transport
**BG01536**	*Beadex (Bx)*	8.9 (0.19)	<0.0001	LIM-only protein 1*	Nervous system development, regulation of transcription from RNA polymerase II promoter
**BG01733**	*CG6175*	9.0 (0.20)	<0.0001	NA	Unknown
**BG01214**	*sugarless (sgl)*	9.2 (0.20)	<0.0001	*UDP-glucose dehydrogenase*	Lipid metabolism, cell surface receptor linked signal transduction
**BG02679** BG00990	*wing blister (wb)*	9.2 (0.15) 7.2 (0.13)	<0.0001 0.0093	*laminin*, alpha 2*	Signal transduction
**BG01127**	*muscleblind (mbl)*	9.6 (0.17)	<0.0001	*muscleblind-like 1*, isoform b*	Peripheral nervous system development, response to stimulus, nucleic acid binding

Lines that survived Bonferroni significance threshold = 0.0011 are indicated in bold font. Human orthologues have homology scores of >0.98 and bootstrap scores of >83% [86]. *Human orthologues associated with known diseases. NA, not applicable.

Our results demonstrate that transcriptional profiling of artificial selection lines is a powerful strategy for identifying genes that contribute to the selected trait, in our case sensitivity to alcohol.

## Discussion

We have used expression microarray analysis to identify genome-wide differences in transcript levels in lines artificially selected for increased resistance or sensitivity to the inebriating effects of ethanol. The realized heritability calculated over 25 generations of selection was modest (approximately 8%). Such heritability is relatively low compared with heritability of locomotor reactivity (approximately 15% [33]) and aggressive behavior (approximately 1% [32]), but it is comparable to realized heritability for mating speed (approximately 7% [31]). There was no correlated phenotypic response for locomotion, aggression, starvation resistance or olfactory behavior, indicating that the response to selection was confined to alcohol sensitivity. This observation agrees with previous reports, in which no significant differences in alcohol sensitivity were observed between lines artificially selected for low and high levels of aggression [32] or for high and low locomotor activity levels [33] from the same base population used in this study.

### *Adh *alleles

We observed differences in Fast and Slow *Adh *allele frequencies between sensitive and resistant lines. However, the probe set for the *Adh *gene was not differentially expressed between the selection lines. This is perhaps not surprising, as previous studies showed that there is no correlation between ethanol tolerance and ADH activity in lines homozygous for the Fast and Slow *Adh *alleles [52] and the increase in tolerance to ethanol in adult flies was not accompanied by an increase in overall ADH activity [42,53,54].

### Whole genome transcriptional profiles of selection lines

Transcriptional profiling studies showed that a large fraction of the genome undergoes altered transcriptional regulation in response to artificial selection, in line with previous selection studies on locomotion, aggression and starvation resistance [32,33,55]. The magnitudes of changes in transcript abundance, although significant at *q *< 0.001, were generally modest. Small (1.3- to 1.4-fold) changes in transcript abundance in response to ethanol exposure have also been reported for other animal models [17]. Similarly, changes in gene expression of as little as 1.4-fold have been detected reproducibly by expression microarray analysis in the brains of human alcoholics [6].

Previously, we observed changes in transcript levels for 582 probe sets after isogenic *Canton S *B flies were exposed to ethanol in an inebriometer [19]. The expression of 195 of these probe sets was also altered between our artificial selection lines (χ_1_^2 ^= 152.1, *p *< 0.0001), including *Adh transcription factor 1*, *Adenylyl cyclase 35C*, 6 *cytochrome P450 *family members, *Glutathione S transferases D5*, *E4*, *E5 *and *E7*, *Heat-shock-protein-70*, *Malic enzyme*, *Neural Lazarillo*, *Pheromone-binding protein-related protein 1*, *2 *and *5*, *Phosphoenolpyruvate carboxykinase*, *Pyruvate dehydrogenase kinase*, and *UDP-glycosyltransferase 35b *(Additional data file 9). One likely reason that we did not detect more of the 582 probe sets previously identified is the difference in genetic background between the two studies (isogenic *Canton S *B versus lines derived from a genetically heterogeneous natural base population).

### Verification of candidate genes

Regardless of whether or not the observed changes in gene expression are causally associated with genetic divergence in alcohol sensitivity between the selection lines, the genes exhibiting altered expression levels are candidate genes affecting alcohol sensitivity. We measured the response to ethanol exposure for 45 mutations in candidate genes that were generated in a common co-isogenic *Canton S *B background, and identified 32 genes with mutational effects on alcohol sensitivity. Three of these genes, *Malic enzyme*, *nuclear fallout *and *longitudinals lacking*, have been previously implicated in alcohol sensitivity and/or tolerance [19] and 23 of them have human orthologues, many of which have been implicated in diseases (Table 2).

The high success rate (73%) of these functional tests supports the hypothesis that expression profiling of genetically divergent lines can identify candidate genes that affect complex traits in *Drosophila *and that comparative genomic approaches can infer human candidate genes from their *Drosophila *orthologues. However, we could not detect genes that are differentially expressed at different developmental times. Similarly, genes affecting the trait that are not regulated at the level of transcription, but may be regulated through posttranslational modifications, will also not be detected by our transcriptional profiling approach.

We determined how many genes that have been already implicated in alcohol sensitivity and/or tolerance in *Drosophila *are significantly differentially expressed between selection lines, and found 38 genes previously implicated in responses to alcohol or alcohol-related metabolism (Additional data file 10). The probe set for *Aldehyde oxidase 1 *[56,57] was not present on the array. Probe sets for the *cheapdate *allele of *amnesiac *[26], the *dopamine D1 receptor *[58] and *neuropeptide F *[59] had absent calls, possibly due to low expression levels, and were consequently not included in the analysis. Of the 34 remaining genes, 10 (approximately 30%) showed altered transcript abundance between our selection lines at *q *< 0.001, including: *Adh *transcriptional factor 1 [60]; *Acetaldehyde dehydrogenase *[61]; *Aldolase *[62]; *fasciclin II*, which is required for the formation of odor memories and for normal sensitivity to alcohol in flies [25]; *Formaldehyde dehydrogenase *[56,57,63]; *geko *[64]; *Glycerol 3 phosphate dehydrogenase *[56,62,63]; and the cell adhesion receptor *slowpoke*, which encodes a large-conductance calcium-activated potassium channel [65,66].

For 14 previously implicated genes (approximately 40%) the magnitude of the differences in expression after selection for alcohol sensitivity and resistance were not great enough to satisfy our stringent false discovery rate threshold of *q *< 0.001 even if the *p *value was < 0.05. Such genes include *dunce *(*q *= 0.07, *p *= 0.03), which encodes a cAMP-phosphodiesterase [26,67]; GABA receptors (*Rdl* and *Lcch3*[68,69]); *lush *(*q *= 0.0031, *p *= 0.009), which encodes an odorant binding protein that interacts with short chain alcohols [70]; the gene that encodes the neuropeptide F receptor (*q *= 0.018, *p *= 0.005) [59]; *period *(*q *= 0.007, *p *= 0.03), a regulator of circadian activity that has been associated with alcohol consumption in mice and humans [28]; *Pka-R1 *(*q *= 0.002, *p *= 0.02) and *Pka-C1 *(*q *= 0.002, *p *= 0.005), which encode a cyclic AMP-dependent protein kinase [27,71]; the calcium/calmodulin-dependent adenylate cyclase encoded by the *rutabaga *gene (*q *= 0.008, *p *= 0.03 [26]); and *sluggish A*, a glutamate biosynthesis enzyme [72].

Expression of only nine alcohol sensitive genes was not significant on our microarray, including: the gene encoding tyramine β-hydroxylase (*p *= 0.23), an enzyme required for the synthesis of octopamine [24,71]; the gene encoding GABA-B receptor-1 (*p *= 0.53) [73]; *hangover *(*p *= 0.52), which encodes a nucleic acid binding zinc finger protein and has been implicated in both the response to heat stress and the induction of ethanol tolerance [24]; and *homer *(*p *= 0.18), which is required for behavioral plasticity [74] - mutant flies exhibit both increased sensitivity to the sedative effects of ethanol and failure to develop normal levels of rapid tolerance [75]. Taken together, around 70% of already implicated genes in alcohol sensitivity were found to be differentially expressed on our microarray.

Other notable probe sets with altered transcriptional regulation include *Sorbitol dehydrogenase 2*, *CG3523*, *CG16935 *and *v(2)k05816*, all of which encode products with alcohol dehydrogenase activity. A previous study reported that mutants in *white rabbit *(*p *= 0.23), which encodes *RhoGAP18B *(*q *= 0.009, *p *= 0.04), are resistant to the sedating effects of ethanol [29]. In our study six probe sets that encode *RhoGap *gene family members (*RhoGAP19D*, *54D*, *16F*, *100F *and *71E*) showed changes in expression levels in response to ethanol selection (*q *< 0.001). Furthermore, 22 of the genes with changes in transcript levels on our microarrays corresponded to genes differentially expressed in the frontal cortex [9] and 10 genes from prefrontal cortex and nucleus accumbens of alcoholics [7] (Additional data file 11).

In addition to human orthologues associated with alcoholism, 246 genes with altered transcript abundance on our microarrays correspond to murine orthologues implicated in altered transcriptional regulation in a meta-analysis study of alcohol drinking preference in mice [3]. *Acetyl Coenzyme A synthase*, *Aldh*, *Beadex*, *CG16935*, *Fdh*, *Laminin B2*, *lethal (2) essential for life *and *lethal(1)G0007 *were among those genes (Additional data file 12). In addition, several *Drosophila *transcripts that are differentially expressed in response to artificial selection have murine orthologues associated with alcohol related phenotypes (Additional data file 12), including *Aldehyde dehydrogenase family 6 member*, which maps to a region on 14q24.23 implicated in alcoholism [76], *Carnitine palmitoyltranferse 1*, *Cathepsin B*, *Distal-less homeobox 1*, *Glutamate oxaloacetate transaminase 2*, *Dorsal switch protein 1 *and *synapsin *[17,77,78].

Flies can readily be grown in large numbers in defined genetic backgrounds under controlled environmental conditions and alcohol sensitivity can be quantified precisely. Our results consolidate the notion that *Drosophila melanogaster *can serve as a gene discovery tool for candidate genes that predispose to alcohol related phenotypes in the human population, and demonstrate the power of transcriptional profiling of selection lines derived from a common base population as a complementary approach for identifying candidate genes for complex traits.

## Materials and methods

### *Drosophila *stocks

Flies were reared on cornmeal/molasses/agar medium under standard culture conditions (25°C, 12:12 hour light/dark cycle). Behavioral assays were conducted in a behavioral chamber (25°C, 70% humidity) between 9 am and 12 am, with the exception of the alcohol selection assays, which were done between 9 am and 2 pm at room temperature. Flies were not exposed to CO_2 _anesthesia for at least 24 hours prior to the assay. Homozygous *P*-element insertion lines containing *P[GT1]*-elements in or near candidate genes in the co-isogenic *Canton S *B background were generated by Dr Hugo Bellen (Baylor College of Medicine, Houston, TX, USA) as part of the Berkeley *Drosophila *Genome Project [50].

### Quantitative assay for alcohol sensitivity

To quantify alcohol sensitivity, we placed flies (*N *= 60-70) in an inebriometer pre-equilibrated with ethanol vapor from which they were eluted at one minute intervals. The MET is a measure of alcohol sensitivity [79].

### Artificial selection for alcohol sensitivity

The base population was generated from 60 isofemale lines established from flies collected in Raleigh, NC in 1999. The isofemale lines were crossed in a round robin design (line 1 ♀ × line 2 ♂, line 2 ♀ × line 3 ♂, …line 60 ♀ × line 1 ♂). Single fertilized females from each cross were placed in each of two culture bottles. In the following generation (G0), the alcohol sensitivity of 60 males and 60 virgin females of each replicate was scored using the alcohol sensitivity assay. The 20 most resistant flies (males and females) from each replicate were placed in bottles to initiate the two resistant lines (R1, R2); and the 20 sensitive flies from each replicate initiated the two sensitive lines (S1, S2). The two control lines were initiated with the remaining 20 flies (C1, C2). In the following (G1) and all subsequent generations, the same procedure was repeated: 60 males and females, separately, from each line (resistant, sensitive, and control) were scored, and the 20 highest-scoring flies from the resistant lines and the 20 lowest-scoring flies from the sensitive lines were selected as parents for the next generation. Control line flies were scored each generation and 20 random flies were used as parents.

Estimates of realized heritability (*h*^2^) were calculated by regression of the cumulative selection response (Σ*R*) on the cumulative selection differential (Σ*S*) [80].

### Correlated responses to selection

To assess the specificity of the selection response, we tested our selected lines for a battery of other traits: locomotor, aggressive, and olfactory behavior, and starvation resistance.

Locomotor behavior was assessed using two different assays. Locomotor reactivity was assessed as described previously [33]. A single three- to five-day-old fly was placed in a vial with approximately 3 ml standard medium, and subjected to gentle mechanical disturbance by tapping on the bench top. The vial was placed horizontally, and the total amount of time the fly remained mobile for a 45 second period immediately following the disturbance was the locomotor reactivity score of the individual. This assay was performed at generation 35, with 20 replicate measurements per line per sex. In the second climbing assay, individual flies were transferred without anesthesia into an empty glass vial, with the height of the vial demarcated in 5 mm intervals from 0 to 27. The fly was tapped to the bottom of the vial, which was then placed vertically. The climbing score was the maximum height reached within the eight second observation period. Twenty replicates per line per sex were tested at generation 36.

Aggressive behavior was assessed as previously described [32]. Aggression of single individuals was quantified by placing one experimental male, with wild-type eye color, with three reference white-eyed isogenic *w*^1118 ^*Canton-S *males. The flies were placed in a vial without food for 90 minutes, after which they were transferred (without anesthesia) to a test arena containing a droplet of food and allowed to acclimate for two minutes. After the acclimation period, the flies were observed for two minutes. The following behaviors were scored as aggressive encounters: kicking, chasing, wing-raising and boxing [81]. The score of the experimental fly was the number of encounters in which it exhibited an aggressive behavior, including interactions initiated by the experimental fly and those in which he responded aggressively to a reference fly. Thirty replicates per line per sex were tested at generation 36.

The olfactory behavior assay was performed as described previously [82]. The flies were placed in a vial without food for 60 minutes and after that were screened by quantifying olfactory avoidance behavior. Five flies per replicate with 15 replicates per sex per line at a concentration of 0.1% (v/v), 0.3% (v/v) and 1% (v/v) benzaldehyde were tested between 9.00 am and 1.00 pm, in a randomized design, in which measurements on individual lines were collected over multiple days to average environmental variation.

Starvation resistance was assessed as previously described [55]. Single sex groups of ten two-day-old flies were placed in vials containing non-nutritive media (1.5% agar and 5 ml water). Survival was scored every eight hours. This assay was conducted at generation 37, with five replicate measurements per line per sex.

### Statistical analysis of correlated responses

Differences between the selection lines for the correlated traits were assessed using a nested mixed model analysis of variance (ANOVA):

*Y *= *μ *+ *Selection *+ *Line *(*Selection*) + *Sex *+ *Selection *× *Sex *+ *Line *(*Selection*) × *Sex *+ *ε*

where *Y *is the phenotypic score, *μ *is the overall mean, *Selection *is the fixed effect of the selection treatment (resistant, control, or sensitive), *Line *(*Selection*) is the random effect of the replicate within each selection group, *Sex *is the fixed effect of sex, and *ε *is the error variance. The terms of most interest in the model are *Selection *and *Line *(*Selection*). A significant *Selection *term is indicative of a correlated response in the trait being tested to selection for alcohol sensitivity. The *Line *(*Selection*) term reveals whether replicate lines responded similarly or divergently, allowing an assessment of the effects of random genetic drift within a replicate line.

### DNA extraction, PCR amplification, restriction digestion

Genomic DNA was extracted from 20 adult males and 20 adult females individually of each selection line using Puregene *DNA purification system *(Gentra systems, Minneapolis, MN, USA). PCR amplification was performed on 100 ng of DNA from each line. The primers were *Adh*-forward 5'CAACATTGGATCCGTCACTG' (1,355 bp) and *Adh*-reverse 5'GCTCAACATCCAACCAGGAG' (1,623 bp). The basic PCR conditions were 35 cycles of denaturation at 94°C for 30 s, annealing at 56°C for 30 s and extension at 72°C for 30 s with 1 unit of *RedTaq *DNA polymerase (Sigma-Aldrich, Carlsband, CA, USA). The 269 bp product was then digested for 3 hours using HpyCH4 IV enzyme (New England BioLabs, Iswich, MA, USA), according to the supplier's instructions. The A-C polymorphism at position 1,490 bp is responsible for the Lys - Thr substitution between Fast and Slow *Adh *alleles [47]. The HpyCH4 IV enzyme recognized the A/CGT nucleotide sequence corresponding to the Slow allele, yielding 240 bp and 29 bp restriction fragments that could be separated by electrophoresis from the 269 bp fragment of the Fast allele.

### Whole genome expression analysis

At generation 25, two replicates of 15 three- to five-day-old virgin males and females were collected from each selection line. Total RNA was extracted from the 24 samples (six lines × two sexes × two replicates) using the Trizol reagent (Gibco BRL, Gaithersburg, MD, USA). Biotinylated cRNA probes were hybridized to high density oligonucleotide microarrays (Affymetrix, Inc. Drosophila GeneChip 2.0) and visualized with a streptavidin-phycoerythrin conjugate, as described in the Affymetrix GeneChip Expression Analysis Technical Manual (2000), using internal references for quantification. The quantitative estimate of expression of each probe set is the *Signal *(*Sig*) metric, as described in the Affymetrix Microarray Suite, Version 5.0.

### Microarray data analysis

The 18,800 probe sets on the Affymetrix Drosophila GeneChip 2.0 are represented by 14 perfect-match (PM) and 14 mismatch (MM) pairs. The *Sig *metric is computed using the weighted log(PM-MM) intensity for each probe set, and was scaled to a median intensity of 500. A detection call of Present, Absent, or Marginal is also reported for each probe set. We excluded probe sets with more than half of the samples called 'Absent' from the analysis, leaving 11,838 probe sets. This filter retained sex-specific transcripts but eliminated probe sets with very low and/or variable expression levels [31]. On the remaining probe sets, we conducted two-way fixed effect ANOVAs of the Signal metric, using the following model:

*Y *= *μ *+ *Line *+ *Sex *+ *Line *× *Sex *+ *ε*

where *Sex *and *Line *are the fixed effects of sex and selection line and *ε *is the variance between replicate arrays. We corrected the *p *values computed in these ANOVAs for multiple tests using a stringent false discovery rate criterion of *q *< 0.001. We used contrast statements [31] to assess whether expression levels of probe sets with *L *and/or *S *× *L *terms at or below the *q *= 0.001 threshold were significantly different between selection groups (resistant, control, and sensitive) at the *p *< 0.05 level, both within each sex and pooled across sexes. All statistical analyses were performed using SAS procedures [29,83]. GO categories were annotated using Affymetrix [84] and FlyBase [85] compilations.

### Functional tests of mutations in candidate genes

We tested whether 37 mutations in 35 of the candidate genes with altered transcript abundance between the selection lines affected alcohol sensitivity. The mutations were homozygous *P[GT1]* elements inserted within the candidate genes, and all were generated in a common co-isogenic background (*Canton S*, B background) [50]. Alcohol sensitivity was assessed for all mutant lines (4-5 replicates/line, *N *= 60-70, 3- to 5-day-old males/replicate) using an inebriometer [79]. We used analysis of variance to assess whether the sensitivity of *P*-element insertion lines differed significantly from the control, according to the model:

Y = *μ *+ *Line *+ *Replicate *(*Line*) + *ε*

where *μ *is the overall mean, *Line *is the fixed effect of line (*P*-element insertion versus Control), *Replicate *is the random effect of replicate, nested within line, and *ε *is the variance within replicates. A significant *Line *term suggests that the mutant is significantly different from the control.

## Abbreviations

*Adh*, *Alcohol dehydrogenase*; C, control line; GO, gene ontology; MET, mean elution time; R, resistant line; S, sensitive line.

## Authors' contributions

TVM, RRHA and TFCM conceived and designed the experiments. TVM performed the experiments. TVM and TFCM analyzed the data. TVM, RRHA and TFCM wrote the paper. All authors read and approved the final manuscript.

## Additional data files

The following additional data are available with the online version of this paper. Additional data file 1 contains a list of probe sets differentially expressed between selection lines at *q *< 0.001. Additional data file 2 contains a list of probe sets with significant differences in contrast statements at *p *< 0.05. Additional data file 3 contains a list of 121 probe sets with larger than two-fold differences in transcript abundance between selection lines. Additional data file 4 contains biological processes GO categories of genes in Additional data file 3. Additional data file 5 contains biological processes GO categories of genes in Additional data file 2. Additional data file 6 contains molecular function GO categories of genes in Additional data file 2. Additional data file 7 contains a list of common probe sets of differentially expressed genes from three artificially selected populations. Additional data file 8 contains biological processes GO categories of genes in Additional data file 7. Additional data file 9 contains a list of common probe sets differentially expressed in response to exposure to ethanol in two experiments (artificial selection for alcohol sensitivity/resistant and tolerance development). Additional data file 10 contains a list of genes previously implicated in alcohol sensitivity in *Drosophila melanogaster*. Additional data file 11 contains a list of *Drosophila *probe sets of genes with human orthologues differentially expressed in alcoholics' brain regions. Additional data file 12 contains a list of *Drosophila *probe sets of genes that are differentially expressed in response to artificial selection and have murine orthologues associated with alcohol related phenotypes.

## Supplementary Material

Additional data file 1 Probe sets differentially expressed between selection lines at *q *< 0.001. Click here for file

Additional data file 2 Probe sets with significant differences in contrast statements at *p *< 0.05. Click here for file

Additional data file 3 The 121 probe sets with larger than two-fold differences in transcript abundance between selection lines. Click here for file

Additional data file 4 Biological processes GO categories of genes in Additional data file 3. Click here for file

Additional data file 5 Biological processes GO categories of genes in Additional data file 2. Click here for file

Additional data file 6 Molecular function GO categories of genes in Additional data file 2. Click here for file

Additional data file 7 Common probe sets of differentially expressed genes from three artificially selected populations. Click here for file

Additional data file 8 Biological processes GO categories of genes in Additional data file 7. Click here for file

Additional data file 9 Common probe sets differentially expressed in response to exposure to ethanol in two experiments (artificial selection for alcohol sensitivity/resistant and tolerance development). Click here for file

Additional data file 10 Genes previously implicated in alcohol sensitivity in *Drosophila melanogaster*. Click here for file

Additional data file 11 *Drosophila *probe sets of genes with human orthologues differentially expressed in alcoholics' brain regions. Click here for file

Additional data file 12 *Drosophila *probe sets of genes that are differentially expressed in response to artificial selection and have murine orthologues associated with alcohol related phenotypes. Click here for file
