# Supplementary material for: Phenotypic and transcriptional response to selection for alcohol sensitivity in Drosophila melanogaster
Source: Genome Biol. 2007 Oct 31;8(10):R231. doi: 10.1186/gb-2007-8-10-r231 (PMC2246305; doi:10.1186/gb-2007-8-10-r231)
Supplement: Additional data file 10 — Genes previously implicated in alcohol sensitivity in Drosophila melanogaster. [file gb-2007-8-10-r231-S10.doc]

**Additional data file 10**. Expression level of the genes previously implicated in alcohol sensitivity in *Drosophila melanogaster*.

| **Probe** | **Gene Name** | **Gene Symbol** | **Absent calls** | **Q>0.001 P>0.05** | **P<0.05 Q>0.001** | **P<0.05**  **Q <0.001** | **References** |
| --- | --- | --- | --- | --- | --- | --- | --- |
| 1641148_a_at | *Acetyl CoA synthetase* | *AcCoAS* |  |  |  | **XX** | [39] |
| 1636020_s_at | *nalyot Adh transcription factor 1* | *Adf1* |  |  |  | **XX** | [60] |
| 1631333_s_at | *alcohol dehydrogenase* | *Adh* |  | XX |  |  | [40, 42-45, 47, 53] |
| 1633473_s_at | *aldolase* | *Ald* |  |  |  | **XX** | [62] |
| 1631993_s_at | *aldolase* | *Ald* |  | XX |  |  | [62] |
|  | *Aldehyde*  *oxidase 1* | *Aldox-1* | not present |  |  |  | [56, 57] |
| 1634096_at | *Acetaldehyde dehydrogenase* | *Aldh* |  |  |  | **XX** | [39, 40, 61] |
| 1625930_at | *cheap date* | *amn* | XX |  |  |  | [26] |
| 1633543_s_at | *Cdc42* | *Cdc42* |  |  | **XX** |  | [29] |
| 1624686_a_at | *dunce* | *dnc* |  |  | **XX** |  | [26] |
| 1629142_at | *dopamine D1 receptor* | *DopR* | XX |  |  |  | [58] |
| 1630160_at | *Fasciclin II* | *Fas2* |  |  |  | **XX** | [25, 26] |
| 1638956_at | *Fasciclin II* | *Fas2* |  |  | **XX** |  | [25, 26] |
| 1640163_at | *Fasciclin II* | *Fas2* |  | XX |  |  | [25, 26] |
| 1624774_a_at | *Fasciclin II* | *Fas2* |  | XX |  |  | [25, 26] |
| 1636740_at | *Formaldehyde dehydrogenase* | *Fdh* |  |  |  | **XX** | [56, 57, 63] |
| 1630047_at | *metabotropic GABA-B receptor subtype 1* | *GABA-B-R1* |  | XX |  |  | [73] |
| 1634113_at | *geko* | *gk* |  |  |  | **XX** | [64] |
| 1636311_at | *Glycerol 3 phosphate dehydrogenase* | *Gpdh* |  |  |  | **XX** | [56, 57, 62] |
| 1616608_a_at | *Glycerol 3 phosphate dehydrogenase* | *Gpdh* |  |  | **XX** |  | [56, 57, 62] |
| 1625949_at | *Glycerol 3 phosphate dehydrogenase* | *Gpdh* |  | XX |  |  | [56, 57, 62] |
| 1634893_at | *Glycerol 3 phosphate dehydrogenase* | *Gpdh* |  | XX |  |  | [56, 57, 62] |
| 1631271_a_at | *hangover* | *hang* |  | XX |  |  | [24] |
| 1634147_a_at | *homer* | *hom* |  | XX |  |  | [75] |
| 1623048_a_at | *Ligand-gated chloride channel homolog 3* | *Lcch3* |  |  | **XX** |  | [68, 69] |
| 1626882_at | *lush* | *lush* |  |  | **XX** |  | [85] |
| 1629644_s_at | *Mig-2-like* | *Mtl* |  |  | **XX** |  | [29] |
| 1629703_at | *neuropeptide F* | *npf* | XX |  |  |  | [59] |
| 1628982_at | *neuropeptide F receptor* | *NPFR1* |  |  | **XX** |  | [59] |
| 1638452_at | *period* | *per* |  |  | **XX** |  | [28] |
| 1632409_a_at | *Protein kinase A* | *Pka-C1* |  |  | **XX** |  | [26, 27, 71] |
| 1631055_at | *Protein kinase A* | *Pka-C1* |  |  | **XX** |  | [26, 27, 71] |
| 1635156_s_at | *cAMP-dependent protein kinase R1* | *Pka-R1* |  |  | **XX** |  | [27, 71] |
| 1625594_s_at | *cAMP-dependent protein kinase type II* | *Pka-R2* |  | XX |  |  | [27, 71] |
| 1627315_s_at | *phospholipase D* | *Pld* |  |  | **XX** |  | [39] |
| 1632279_at | *glass multimer reporter construct of Nolan* | *Rac1* |  | XX |  |  | [29] |
| 1624321_at | *glass multimer reporter construct of Nolan* | *Rac2* |  |  |  | **XX** | [29] |
| 1635005_a_at | *Resistance to dieldrin* | *Rdl* |  |  | **XX** |  | [68, 69] |
| 1626808_s_at | *glass multimer reporter construct of Hariharan* | *Rho1* |  | XX |  |  | [29] |
| 1625488_s_at | *glass multimer reporter construct of Hariharan* | *Rho1* |  | XX |  |  | [29] |
| 1634481_at | *RhoGAP18B* | *RhoGAP18B* |  |  | **XX** |  | [29] |
| 1628594_at | *rutabaga* | *rut* |  |  | **XX** |  | [26] |
| 1640090_a_at | *sluggish A* | *slg* |  |  | **XX** |  | [72] |
| 1637301_a_at | *slowpoke* | *slo* |  |  |  | **XX** | [65, 66] |
| 1627317_a_at | *tyramine-beta-hydroxylase* | *Tbh* |  | XX |  |  | [20, 24, 71] |
| 1629859_s_at | *vacuolar peduncle* | *vap* |  | XX |  |  | [20, 26] |
| 1630866_at | *CG7502* | *whir* |  | XX |  |  | [29] |
